# Supplementary material for: Algorithm-assisted interpretation of cyclic and differential pulse voltammetry for cardiac troponin detection
Source: PLoS One. 2026 May 13;21(5):e0348348. doi: 10.1371/journal.pone.0348348 (PMC13170963; doi:10.1371/journal.pone.0348348)
Supplement: Attachment — Submitted filename: PONE-D-24-56032-reviewed.pdf [file pone.0348348.s001.pdf]

PLOS ONE

Interactive Graphical Visualization For Electrochemical Voltammetry Techniques of Differential Pulse Voltammetry and Cyclic Voltammetry

--Manuscript Draft--

**Manuscript Number:**	PONE-D-24-56032
**Article Type:**	Research Article
**Full Title:**	Interactive Graphical Visualization For Electrochemical Voltammetry Techniques of Differential Pulse Voltammetry and Cyclic Voltammetry
**Short Title:**	Interactive Graphical Visualization For Electrochemical Voltammetry Techniques
**Corresponding Author:**	Wikan Danar Sunindyo, Dr.<br>Bandung Institute of Technology: Institut Teknologi Bandung<br>BANDUNG, WEST JAVA INDONESIA
**Keywords:**	software; frontend; backend; measurement data analysis; baseline; peak; voltammetry; heart disease
**Abstract:**	The potential for heart disease can be detected using a sensor reader that employs electrochemical voltammetry techniques and allows for the rapid identification of critical biomarkers, such as cardiac troponin, producing raw voltammetry test data. This research focuses on developing an interactive graphical visualization of heart disease detection monitoring system capable of processing raw data from voltammetry measurements into easily interpretable information regarding heart disease potential. The process involves converting complex data into a user-friendly format accessible through a web interface. The backend system implementation utilizes FastAPI technology and a PostgreSQL database to manage data and communicate with the client side. System testing and evaluation were conducted through various functional and performance scenarios of the backend application. The testing results of the backend system architecture demonstrate reliability and effectiveness in processing and presenting data as per the defined requirements
**Order of Authors:**	Wikan Danar Sunindyo, Dr.
	Kristo Abdi Wiguna
	Marcelus Michael Herman Kahari
	Uperianti Uperianti, S.T.
	Isa Anshori, Ph.D.
	Infall Syafalni, Dr.
	Latifa Dwiyanti, M.T.
**Additional Information:**	
**Question**	**Response**
**Financial Disclosure**<br><br>Enter a financial disclosure statement that describes the sources of funding for the work included in this submission. Review the submission guidelines for detailed requirements. View published research articles from *PLOS ONE* for specific examples.<br><br>This statement is required for submission and **will appear in the published article** if	Yes

Powered by Editorial Manager® and ProduXion Manager® from Aries Systems Corporation

the submission is accepted. Please make sure it is accurate.<br><br>**Funded studies**<br>Enter a statement with the following details:<br>• Initials of the authors who received each award<br>• Grant numbers awarded to each author<br>• The full name of each funder<br>• URL of each funder website<br>• Did the sponsors or funders play any role in the study design, data collection and analysis, decision to publish, or preparation of the manuscript?<br><br>Did you receive funding for this work?	
Please add funding details.<br>as follow-up to "**Financial Disclosure**<br><br>Enter a financial disclosure statement that describes the sources of funding for the work included in this submission. Review the submission guidelines for detailed requirements. View published research articles from *PLOS ONE* for specific examples.<br><br>This statement is required for submission and **will appear in the published article** if the submission is accepted. Please make sure it is accurate.<br><br>**Funded studies**<br>Enter a statement with the following details:<br>• Initials of the authors who received each award<br>• Grant numbers awarded to each author<br>• The full name of each funder<br>• URL of each funder website<br>• Did the sponsors or funders play any role in the study design, data collection and analysis, decision to publish, or preparation of the manuscript?<br><br>Did you receive funding for this work?"	The funding is from P2MI 2024 Program of School of Electrical Engineering and Informatics Institut Teknologi Bandung
Please select the country of your main	INDONESIA - ID

research funder (please select carefully as in some cases this is used in fee calculation).<br>as follow-up to "**Financial Disclosure**<br><br>Enter a financial disclosure statement that describes the sources of funding for the work included in this submission. Review the submission guidelines for detailed requirements. View published research articles from *PLOS ONE* for specific examples.<br><br>This statement is required for submission and **will appear in the published article** if the submission is accepted. Please make sure it is accurate.<br><br>**Funded studies**<br>Enter a statement with the following details:<br>• Initials of the authors who received each award<br>• Grant numbers awarded to each author<br>• The full name of each funder<br>• URL of each funder website<br>• Did the sponsors or funders play any role in the study design, data collection and analysis, decision to publish, or preparation of the manuscript?<br><br>Did you receive funding for this work?"	
**Competing Interests**<br><br>Use the instructions below to enter a competing interest statement for this submission. On behalf of all authors, disclose any competing interests that could be perceived to bias this work—acknowledging all financial support and any other relevant financial or non-financial competing interests.<br><br>This statement is required for submission and **will appear in the published article** if the submission is accepted. Please make sure it is accurate and that any funding sources listed in your Funding Information	The authors have declared that no competing interests exist.

later in the submission form are also declared in your Financial Disclosure statement.<br><br>View published research articles from *PLOS ONE* for specific examples.<br><br>**NO authors have competing interests**<br><br>Enter: *The authors have declared that no competing interests exist.*<br><br>**Authors with competing interests**<br><br>Enter competing interest details beginning with this statement:<br><br>*I have read the journal's policy and the authors of this manuscript have the following competing interests: [insert competing interests here]*<br><br>* typeset	
**Ethics Statement**<br><br>Enter an ethics statement for this submission. This statement is required if the study involved:<br><br>• Human participants<br>• Human specimens or tissue<br>• Vertebrate animals or cephalopods<br>• Vertebrate embryos or tissues<br>• Field research<br><br>Write "N/A" if the submission does not require an ethics statement.<br><br>General guidance is provided below. Consult the submission guidelines for detailed instructions. **Make sure that all information entered here is included in the Methods section of the manuscript.**	N/A

**Format for specific study types**<br><br>**Human Subject Research (involving human participants and/or tissue)**<br>• Give the name of the institutional review board or ethics committee that approved the study<br>• Include the approval number and/or a statement indicating approval of this research<br>• Indicate the form of consent obtained (written/oral) or the reason that consent was not obtained (e.g. the data were analyzed anonymously)<br><br>**Animal Research (involving vertebrate animals, embryos or tissues)**<br>• Provide the name of the Institutional Animal Care and Use Committee (IACUC) or other relevant ethics board that reviewed the study protocol, and indicate whether they approved this research or granted a formal waiver of ethical approval<br>• Include an approval number if one was obtained<br>• If the study involved *non-human primates*, add *additional details* about animal welfare and steps taken to ameliorate suffering<br>• If anesthesia, euthanasia, or any kind of animal sacrifice is part of the study, include briefly which substances and/or methods were applied<br><br>**Field Research**<br><br>Include the following details if this study involves the collection of plant, animal, or other materials from a natural setting:<br>• Field permit number<br>• Name of the institution or relevant body that granted permission	
**Data Availability**<br><br>Authors are required to make all data underlying the findings described fully available, without restriction, and from the time of publication. PLOS allows rare exceptions to address legal and ethical concerns. See the PLOS Data Policy and FAQ for detailed information.	Yes - all data are fully available without restriction

Powered by Editorial Manager® and ProduXion Manager® from Aries Systems Corporation

A Data Availability Statement describing where the data can be found is required at submission. Your answers to this question constitute the Data Availability Statement and **will be published in the article**, if accepted.<br><br>**Important:** Stating 'data available on request from the author' is not sufficient. If your data are only available upon request, select 'No' for the first question and explain your exceptional situation in the text box.<br><br>Do the authors confirm that all data underlying the findings described in their manuscript are fully available without restriction?	
**Describe where the data may be found in full sentences. If you are copying our sample text, replace any instances of XXX with the appropriate details.**<br><br>• If the data are **held or will be held in a public repository**, include URLs, accession numbers or DOIs. If this information will only be available after acceptance, indicate this by ticking the box below. For example: *All XXX files are available from the XXX database (accession number(s) XXX, XXX.*).<br>• If the data are all contained **within the manuscript and/or Supporting Information files**, enter the following: *All relevant data are within the manuscript and its Supporting Information files.*<br>• If neither of these applies but you are able to provide **details of access elsewhere**, with or without limitations, please do so. For example:<br><br>*Data cannot be shared publicly because of [XXX]. Data are available from the XXX Institutional Data Access / Ethics Committee (contact via XXX) for researchers who meet the criteria for access to confidential data.*<br><br>*The data underlying the results presented in the study are available from (include the name of the third party*	this information will only be available after acceptance

and contact information or URL).<br>• This text is appropriate if the data are owned by a third party and authors do not have permission to share the data.<br><br>* typeset	
Additional data availability information:	Tick here if the URLs/accession numbers/DOIs will be available only after acceptance of the manuscript for publication so that we can ensure their inclusion before publication.

Powered by Editorial Manager® and ProduXion Manager® from Aries Systems Corporation

Manuscript

Click here to access/download;Manuscript;Web App Cardiac Sensor_Manuscript 241204.docx

Interactive Graphical Visualization For Electrochemical Voltammetry Techniques of Differential Pulse Voltammetry and Cyclic Voltammetry

Author Names:

Wikan Danar Sunindyo, Kristo Abdi Wiguna, Marcelus Michael Herman Kahari, Uperianti, Isa Anshori, Infall Syafalni, Latifa Dwiyanti

School of Electrical Engineering and Informatics, Bandung Institute of Technology, Jl. Ganesa No.10, Lb. Siliwangi, Coblong, Bandung, Jawa Barat 40132

Abstract

The potential for heart disease can be detected using a sensor reader that employs electrochemical voltammetry techniques and allows for the rapid identification of critical biomarkers, such as cardiac troponin, producing raw voltammetry test data. This research focuses on developing an interactive graphical visualization of heart disease ~~detection~~ monitoring system capable of processing raw data from voltammetry measurements into easily interpretable information regarding heart disease potential. The process involves converting complex data into a user-friendly format accessible through a web interface. The backend system implementation utilizes FastAPI technology and a PostgreSQL database to manage data and communicate with the client side. System testing and evaluation were conducted through various functional and performance scenarios of the backend application. The testing results of the backend system architecture demonstrate reliability and effectiveness in processing and presenting data as per the defined requirements

Keywords: *software, frontend, backend, measurement data analysis, baseline, peak, voltammetry, heart disease*

1. Introduction

Heart disease is often cited as one of the leading causes of death in many countries. According to the World Health Organization (WHO), approximately 18 million people died from heart disease in 2019, accounting for 32% of all deaths worldwide [1]. As the number one cause of death globally, heart disease presents a significant challenge in the healthcare sector. Early detection is one of the key strategies for preventing heart disease. Various methods can be employed to detect heart disease ~~at an~~ early stage, ~~one of which involves~~ using voltammetry measurements on blood. Voltammetry is an electroanalytical method that provides information about an analyte, the substance being analyzed, through the measurement process. In this context, the voltammetry method is used alongside a potentiostat to identify the presence of the analyte cardiac troponin in the blood, ~~which is~~ a crucial indicator in diagnosing heart disease.

Previous research has successfully utilized potentiostats to detect cardiac troponin in blood, resulting in the production of raw data. However, to date, there is no system specifically designed to process this raw data into easily understandable and interpretable information. Consequently, laboratory technicians and the public interested in gaining insights from this data face significant

challenges. Without an appropriate system, the raw data remains a collection of numbers and graphs that are difficult to interpret. This highlights an urgent need for the development of a system capable of translating voltammetry data into clear and accessible information, thereby facilitating early detection of heart disease.

To further enhance this system, it is essential to integrate a cloud service that can interface directly with the sensor reader. By utilizing cloud-based technology, data from the electrochemical sensors used in voltammetry can be collected, stored, and accessed in real-time. This allows for the continuous monitoring of patient data, providing healthcare professionals with timely and remote access to critical information necessary for early diagnosis.

Moreover, there is a significant need for processing automation that converts raw data from these electrochemical sensors into actionable insights. The proposed system should automate the entire data processing pipeline—from data acquisition to the final diagnostic output—thereby reducing human error, speeding up the analysis process, and enabling more precise and reliable results.

Finally, the core data processed by this system will originate from electrochemical sensors, which play a pivotal role in detecting specific analytes such as cardiac troponin. These sensors generate complex electrochemical signals that require sophisticated algorithms to accurately interpret. By automating the interpretation of these signals, the system can produce a clear, concise diagnosis, making the early detection of heart disease more accessible and efficient.

Therefore, there is a requirement for a system that can serve as a central hub for processing the results of voltammetry measurements and analyzing this measurement data into interpretable and understandable information. The system will also assist in automating the verification process. This system will take the form of web-based software, integrated with cloud services for sensor data management, and designed to fully automate the processing of electrochemical sensor data into actionable insights for early heart disease detection.

2. Materials and Methods

2.1. Potentiostat & Voltammetry

2.1.1 Potentiostat

The potentiostat is a tool analytics designed to control the potential of the working electrode in the cell electrochemistry with several other electrodes [2]. Potentiostat will control the balance of diffusion current on the working electrode with transfer electrons in the opposite direction. If a reduction reaction occurs on the working electrode, an oxidation reaction occurs on the auxiliary electrode.

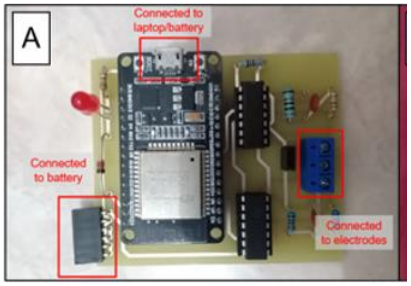

Fig. 1. Example of potentiostat hardware

2.1.2 Voltammetry

Voltammetry is an electroanalytical method in which information about the analyte (substance to be studied) is obtained from the process of measuring current as a function of the applied potential. This method is based on electrochemical concepts. The time function results from applying electrical voltage to the working electrode and comparison electrode and the current profile. The current measured is a diffusion current where the appearance of the current is due to the oxidation-reduction process (redox reaction) of the electroactive analyte on the electrode surface. [4]

2.1.2.1. CV

Cyclic Voltammetry (CV) is a popular method in electrochemistry which is used to carry out analysis of reduction and oxidation processes in analytes. CV is also very important for studying electron transfer-initiated chemical reaction processes, including catalysis. [5]

How CV works is as follows. The potentiostat will take potential readings on the working electrode and compare electrodes linearly until it reaches the point that has been determined as the limit point. After reaching the limit point, the reading is taken again in the opposite direction. This process will be carried out repeatedly for a few moments and the change in current between the working electrode and the comparator is measured. The results of this reading will be formed as a characteristic graph known as a cyclic and duck-shaped voltammogram.

2.2.2.2. DPV

Differential Pulse Voltammetry (DPV) is a pulse technique that is designed to minimize background charging currents. [6] This method is very good for measuring analytes at relatively low concentrations for organic compounds or inorganic compounds. This method is a derivative of the Normal Pulse Voltammetry (NPV) method.

In the DPV method, a potential value can be selected for a reaction where there is no faradaic reaction and applied to the electrode. The basic potential is increased between pulses at the same rate of increase. Current measurements are made before and after pulse application and the difference between the two measurements is recorded.

2.2. Sensor Reader and Data Analysis

2.2.1 Sensor Reader

The sensor reader utilized in this research is based on previous work by Anshori, titled “Design of a Smartphone-Controlled Low-Cost Potentiostat for Cyclic Voltammetry Analysis Based on the ESP32 Microcontroller” [3] and has been specifically adjusted for heart disease detection using voltammetry, an electrochemical technique to measure the presence of cardiac biomarkers in blood samples. The primary instrument in this process is a potentiostat, which plays a critical role in controlling the voltage applied to the electrochemical cell, comprising working, reference, and auxiliary electrodes. When a blood sample is introduced into the system, the potentiostat applies a varying potential to the working electrode, initiating a redox reaction in the presence of the target analyte—cardiac troponin in this case—producing an electric current proportional to the analyte's concentration.

The resulting current, which is the raw data, is then recorded over a range of applied potentials. This raw data, presented as a voltammogram (a plot of current versus potential), is the foundation for further analysis. The sensor reader captures these signals and transmits them to the backend system for detailed processing. The data captured by the sensor reader needs to be carefully processed to identify critical points such as the baseline and peak signals, which are essential for interpreting the presence and concentration of cardiac biomarkers.

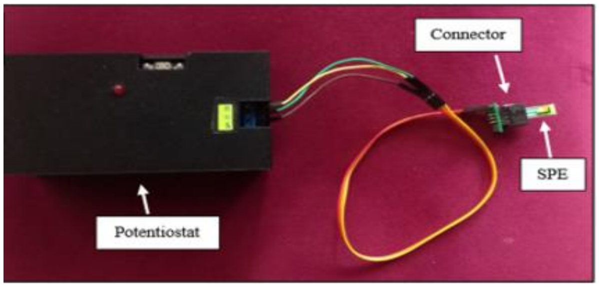

Fig. 2. Potensiostat hardware used for sensor reader

2.2.2 Input Data Representation

The following figures illustrate the raw data format obtained from measurements conducted using a potentiostat with cyclic voltammetry (CV) and differential pulse voltammetry (DPV) methods.

V,µA,V,µA,V,µA
0,18.0879,0,1.20399,0,0.433998
-0.0102076,14.2799,-0.0102076,0.328998,-0.0102076,-0.356998
-0.0204151,11.144,-0.0204151,-0.531997,-0.0204151,-1.17599
-0.0306227,8.39996,-0.0306227,-1.37899,-0.0306227,-2.05799
-0.0408303,5.99197,-0.0408303,-2.24699,-0.0408303,-3.14299
-0.0510379,3.80798,-0.0510379,-3.19549,-0.0510379,-4.84398
-0.0612454,1.95999,-0.0612454,-4.64798,-0.0612454,-7.90297
-0.071453,0.447998,-0.071453,-7.80497,-0.071453,-12.5229
-0.0816606,-0.727996,-0.0816606,-13.8599,-0.0816606,-18.8964
-0.0918681,-1.63799,-0.0918681,-22.3754,-0.0918681,-27.0444
-0.102075,-2.32399,-0.102075,-31.2759,-0.102075,-35.6928
-0.112283,-2.83499,-0.112283,-39.9978,-0.112283,-44.1208
-0.12249,-3.21299,-0.12249,-48.4258,-0.12249,-52.1428
-0.132698,-3.52798,-0.132698,-56.1537,-0.132698,-59.6257
-0.142905,-3.80798,-0.142905,-62.9017,-0.142905,-66.4997
-0.153113,-4.03198,-0.153113,-68.4597,-0.153113,-72.6457
-0.163321,-4.19998,-0.163321,-72.7857,-0.163321,-77.5737
-0.173528,-4.36098,-0.173528,-75.2917,-0.173528,-80.4156

Fig. 3. Raw data format of the measurement result based on voltametry using CV method

For CV, there are three data sets, each with two columns representing voltage (X-axis) and current (Y-axis). The second data set is used for analysis as it provides the most accurate reference when sensor readings are stable. Peak information is determined by identifying the highest current (Y-axis), while a specialized algorithm is required to correct the baseline due to signal variability.

V,µA
-0.999804,170.239
-0.989597,170.351
-0.979389,152.655
-0.969181,145.543
-0.958974,142.463
-0.948766,137.311
-0.938559,132.495
-0.928351,129.303
-0.918144,127.343
-0.907936,126.447
-0.897728,125.439
-0.887521,124.319
-0.877313,124.375
-0.867106,123.983
-0.856898,123.311
-0.846691,123.087
-0.836483,123.143

Fig. 4. Raw data format of the measurement result based on voltametry using DPV method

For DPV, the raw data format consists of a single data set with two columns for voltage (X-axis) and current (Y-axis). Similarly, peak information is derived by finding the highest current value, and a special algorithm is needed to correct the baseline due to varying signals. This information will be used to calculate the distance from the peak to the baseline, which in turn is used to determine the current response value when compared with other raw measurement data files.

However, working directly with these raw data sets, provided as CSV files containing numerous numerical values, makes it difficult to visually detect or interpret patterns. The sheer amount of numerical data is hard to read and analyze manually, making it challenging to identify critical

peaks and baselines without specialized algorithms and tools for data visualization and analysis. Therefore, there is a need to analyze these numbers using specialized software and algorithms to accurately interpret the data, identify peak signals, and differentiate them from the baseline, as manual interpretation of such extensive numerical data is not feasible.

2.2.3 Data Analysis

The analysis of voltammetry data revolves around two critical components: the baseline and the peak signal. The baseline represents the stable, non-responsive current when no electrochemical reaction is occurring. It essentially provides a reference level against which changes in current can be measured. In contrast, the peak signal corresponds to the current spike observed during an electrochemical reaction, typically resulting from the redox processes of the analyte.

The difference between the baseline and peak signal is that the baseline remains relatively constant and unaffected by the presence of analytes, while the peak signal reflects the presence and concentration of the target analyte as it undergoes oxidation or reduction.

The use of these components are fundamental to the interpretation of voltammetry data: the baseline allows for the correction of background noise, ensuring that the measured signal is attributed solely to the analyte. The peak signal, on the other hand, provides quantitative information about the analyte’s concentration, with higher peaks generally indicating greater concentrations of the analyte in the sample. Accurate identification and correction of the baseline are crucial to reliably measure the peak signal and, consequently, the concentration of the analyte.

During the data analysis process, the system must first establish a baseline by identifying regions in the voltammogram where no significant electrochemical activity occurs. This is achieved through algorithms that correct for any drift or noise in the baseline, ensuring that subsequent peak measurements are accurate. Once the baseline is established, the system then focuses on identifying the peak signals, which correspond to the maximum current observed during the analyte's oxidation or reduction.

For differential pulse voltammetry (DPV), peaks are identified at specific potentials where the pulse modulates the current response. The peak height, which is the difference between the baseline and the peak current, directly correlates with the concentration of the analyte—in this case, cardiac troponin [7]. Similarly, in cyclic voltammetry (CV), peaks are observed during both the forward and reverse scans, with the current peak representing the oxidation and reduction of the analyte.

The system's backend processes these peaks to determine the analyte concentration, which is crucial for diagnosing heart disease. By comparing the peak heights and their corresponding baseline values across different samples, the system can quantify the presence of cardiac biomarkers, thus enabling early detection of heart disease. This automated analysis significantly reduces the need for manual interpretation and ensures that the results are both accurate and reliable.

2.3. Software for Sensor Reader

The data obtained from electrochemical voltammetry techniques using a potentiostat for detecting cardiac troponin in samples needs to be processed for displaying it to the user. This section discusses the software for the sensor reader.

2.3.1. Requirement System

2.3.1.1. Flowchart

First, users need to input their blood sample in the Potensiostat device. After that, the Potensiostat will read and process the input and return a txt file that contains the current mA and potential V. After users receive the file, users need to input the file to the website. Website will send the file to the backend system and the whole processing will happen in the backend. After the backend finishes, the website will receive the result. If the user is admin, they can also see the graphical output from the Potensiostat device. If not, users only see the result that their potential of heart disease is low or high.

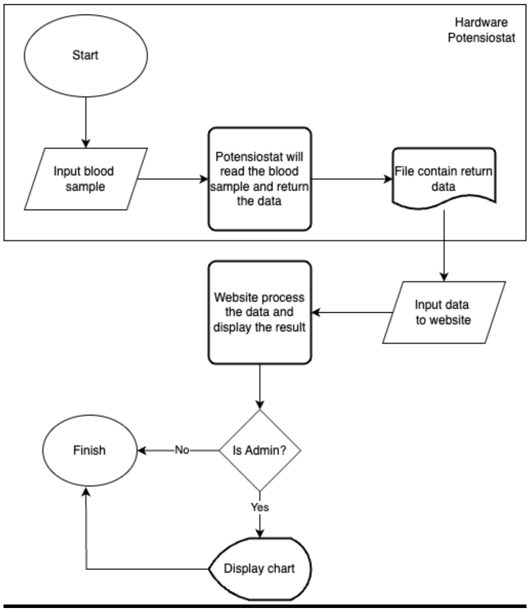

Fig. 5. Flowchart diagram of software system

2.3.1.2. Functional Requirement

Based on the flowchart diagram, we create the list of functional requirements that need to be fulfilled during the development of the program.

Table. 1. Functional requirement of software system

ID	Requirement	Description
F01	The system should accept user information from users who want to create an account.	Users can create an account by providing an email and password.

F02	The system should accept login information from users who want to sign in.	Users can log in using their email and password.
F03	The system should accept potentiostat data from users through the website.	Users can upload potentiostat data to the website.
F04	The system should display predictions of heart disease potential for users.	Users can view the predicted potential for heart disease based on data obtained from a potentiostat device.
F05	The system should allow changing a regular account to a lab technician account and vice versa.	Admins can convert a regular account to a lab technician account and vice versa on the designated page.
F06	The system should display the test history for each user.	Regular users can view the history and results of tests they have conducted.
F07	For lab technicians and admins, the system should display the test history of all users.	Lab technicians and admins have access to view the test history of all users.
F08	The system should display graphical visualizations of data captured by the potentiostat device.	Lab technicians and admins can view and analyze graphical visualizations of data captured by the device.

2.3.2. Architecture System

Based on the requirement system that was proposed in 2.3.1, we decided to create several components for the frontend system. The component will work and connect with the backend system to process the input data from the user. The green component in the figure below represents the component that was made by the author. In contrast, the red component represents the component that was imported from others library, for example Recharts and Plotly which is the open source library for displaying graph.

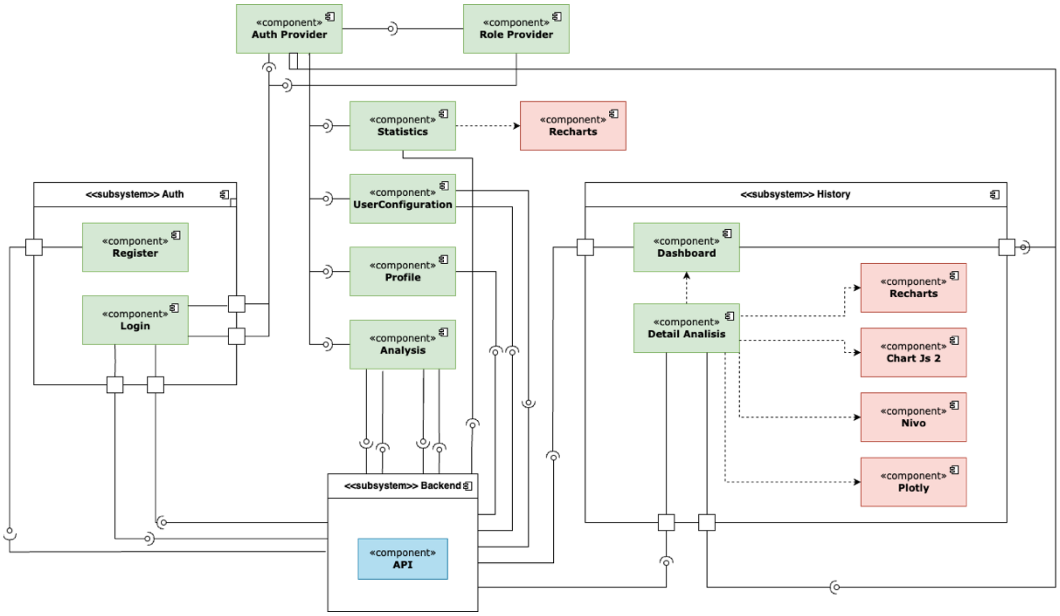

Fig. 6. Component diagram for frontend system

2.3.3. Backend System

The backend system for the heart disease detection software is designed with a focus on efficiency, scalability, and reliability. It integrates several key technologies to manage data processing, storage, and communication with the frontend.

The backend utilizes a REST API to facilitate communication between the client-side application and the server. REST API is chosen for its simplicity, scalability, and compatibility with web and mobile applications. It provides stateless endpoints for tasks like uploading measurement data and retrieving analysis results, ensuring smooth interaction between the frontend and backend. PostgreSQL is employed as the relational database management system for storing structured data, including user information, measurement data, and analysis results. Known for its robustness and support for ACID properties, PostgreSQL ensures data integrity and efficient query handling, making it ideal for the backend's needs.

The backend is developed using FastAPI, a modern, high-performance web framework for building APIs. FastAPI's support for asynchronous operations enables efficient handling of concurrent data processing tasks, ensuring the system remains responsive even under heavy load. Google Cloud Storage is integrated for managing the large volumes of data generated by voltammetry measurements. It offers scalable and secure storage solutions, allowing the backend to handle extensive datasets without burdening local resources, and ensuring easy access to data from any location.

2.3.4. Frontend System

A frontend framework can be defined as a toolkit used by developers to build a website interface. With a framework, developers can organize their code more effectively and develop faster.

Additionally, frameworks help create a more visually appealing, responsive, and efficient user experience.

An example of a frontend framework is React. Each framework has its own unique approach and features. These frameworks provide ready-to-use development components, efficient state management, well-managed routing capabilities, and smooth integration processes with the server side. For this software development, React will be used as the system framework. For styling, we will use Tailwind as a CSS framework.

2.3.4.1. Chart

Data visualization is the representation and presentation of data that uses visual perception abilities to enhance cognition or the process of gaining knowledge [8]. Creating visualizations must be done carefully to avoid causing misperceptions for users when interpreting the data.

In presenting data, several supporting aspects are involved concerning the design features to be used in the data visualization. Decisions regarding the implementation of design features must consider the additional meaning users might gain when reading the data visualization, how intuitive it is, and the depth of knowledge obtained by the readers.

Chart is part of important things for laborent and admin when seeing the chart of analysis results. They can check if there are any anomaly in the data or if the program gives the wrong result. Also, there will be some cases that the output of the analysis is wrong because of several reasons, like a little error or another.

2.3.4.1.1. Annotation

Annotation is crucial because it helps readers understand a data visualization [8]. One form of annotation is visual annotation, which uses reference lines. The use of reference lines can assist readers in understanding the key data points that the creator of the visualization wants to highlight.

2.3.4.1.2. Interactivity

With the advancement of technology, interactivity can enhance the user experience in reading data visualizations. One form of interactivity is annotated details. When users hover over a point on a chart, an extra layer can be displayed showing details of the data. This can increase the reader's confidence in accurately interpreting the data in detail.

Another form of interactivity is manipulating parameters. By manipulating parameters, users can view various representations of the data. For example, in Differential Pulse Voltammetry (DPV), if users want to view only the sample graph and remove the benchmark, they can apply filters, making it easier for users to represent the data.

2.3.4.2. Chart Library

2.3.4.2.1. Recharts

Recharts is a library for displaying charts built using React and D3. This library utilizes D3 to create high-performance and interactive SVG visualizations. It is also easy to customize according to user needs. Recharts is an open-source library.

2.3.4.2.2. Chart Js

Chart.js is an open-source library and one of the most popular libraries among JavaScript developers, with approximately 2.4 million downloads per week based on NPM downloads and around sixty thousand GitHub stars. Created in 2013, it is licensed under the MIT License.

2.3.4.2.3. Nivo

Nivo is a library built on React components to assist developers in creating data visualizations. It is developed on top of D3 and is open-source, maintained by the community.

2.3.4.2.4. Plotly

Plotly is an open-source library developed by Plotly Technologies Inc. It is built on top of D3 and Stack.gl. Plotly is a high-level library that allows users to easily create data visualizations in various forms.

3. Results & Discussion

The proposed capstone solution includes the development of a backend system that communicates with the frontend system via the HTTP protocol. The backend system will be deployed on Google Cloud Platform and frontend system will be deployed on Netflify. The software being developed is a web application intended for healthcare professionals such as doctors and lab technicians, as well as the public using computers as operational devices. The backend service will receive raw data files from users obtained through voltammetry techniques, specifically cyclic voltammetry or differential pulse voltammetry. This raw data will be analyzed to produce information that the client will receive, specifically predictions of potential heart disease.

To obtain this information, two raw data files with DPV configuration are required, along with the search for current response values. One file serves as a benchmark sample, and the other as a sample that has been treated with antigens, either healthy or diseased. The current response value is obtained by calculating the difference between the peak-to-baseline distance of the two raw data measurements. This necessitates the identification of baselines and peaks for each raw data measurement. The solution to these problems is realized through software analysis and design aimed at supporting users in achieving their goals within the backend aspect of the application.

3.1. Voltammetry Data Analysis

To analyze raw voltammetry data, it is essential to identify the baseline and peak locations to measure the distance between them. Baseline correction is needed to remove noise and achieve accurate analysis. Several algorithms, such as polynomial fit, gradient, adaptively reweighted penalized least squares, adaptively iteratively reweighted penalized least squares, and asymmetric least squares, will be tested for optimal baseline correction using software like PSTrace. Peak locations will be identified using Python libraries, enabling the calculation of the peak-to-baseline

distance. For heart disease prediction, the current response values from two DPV measurement files will be subtracted.

The analysis results of the raw data will be stored in JSON format, including information such as current, voltage, peak location, baseline, and the distance between the baseline and peak. This information will be stored in the attributes `analysis_1` or `analysis_2`. Below are examples of the analysis results stored in JSON format as depicted in figures below.

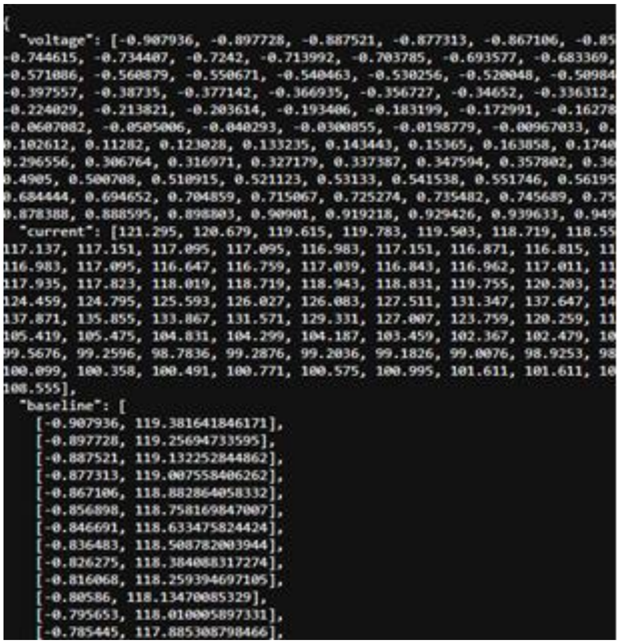

Fig. 7. Result on analysis on the measured data (1)

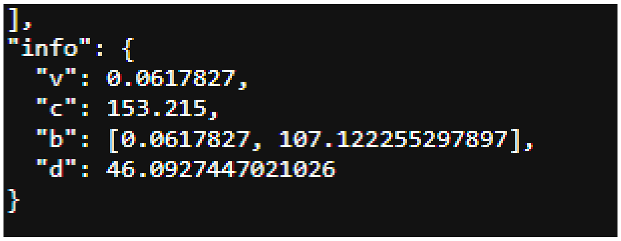

Fig. 8. Result on analysis on the measured data (2)

To predict the user's potential for heart disease, a raw data file from voltammetry measurements using the DPV configuration and its analysis results, which include the current response value, are needed. The current response value will be compared to the DPV method's threshold. The information obtained from the voltammetry data analysis is received by the client and represented as a curve with the analysis results. In this analysis, v is the voltage coordinate for the peak location, c is the current coordinate for the peak location, b is the location where the peak is vertically placed on the baseline detected, and d is the distance between b and the peak location called current response.

The flow for predicting a user's potential for heart disease is as follows: if the current response value is less than the threshold, the prediction will be negative, indicating a low potential for heart disease. Conversely, if the current response value is greater than the threshold, the prediction will be positive, indicating a high potential for heart disease. The analysis results will update the `result` attribute in the Measurement data model. If the potential for heart disease is high, the `result` attribute will be `true`; if low, it will be `false`. Additionally, the current response value will be added to the `current_response` attribute of the Measurement data model.

Below is a comparison table mapping current response values and heart disease potential predictions based on the implemented backend system:

Table 2. Comparison for threshold and current response

**Comparison**	**Disease Likelihood**	**Result**
Current response > threshold	High potential	True
Current response <= threshold	Low potential	False

3.1.1. Database Design

Based on the problem explained, it can be concluded that each measurement involves raw data files from voltammetry techniques and their corresponding analysis results, including heart disease risk predictions for specific users with CV or DPV configurations and certain thresholds. As a solution, a data modeling schema is created using an entity-relationship diagram (ERD), as shown in figure below. SQL database was chosen for its suitability in handling structured data.

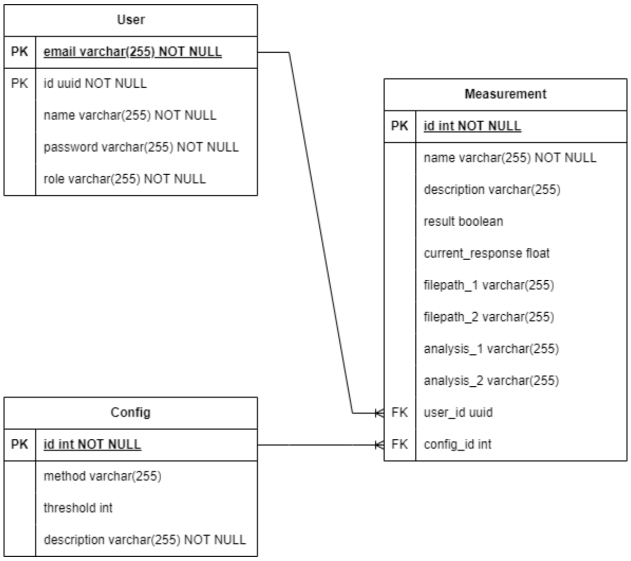

Fig. 9. Database schema

CSV files will be stored in the designated filepath attributes. For cyclic voltammetry (CV), a single CSV file will be generated and saved in `filepath_1`, which contains the data required for analysis. In the case of differential pulse voltammetry (DPV), two CSV files are necessary: both files will be saved in `filepath_1`. This dual-file setup is critical for conducting comparative analysis between the baseline data and the measured data, which helps determine the presence or absence of the disease. The results of the analysis will be stored in `analysis_1` for the evaluation of data from `filepath_1`, and in `analysis_2` for the analysis of additional files in `filepath_2`, ensuring a robust and comprehensive interpretation of the collected data. The result of the comparison will be saved into the result field to indicate whether the data meets the diagnostic criteria by using the threshold number field in the config model.

3.2. Backend System

This application is designed as a web application and aims to efficiently process and analyze data accurately. Selecting the appropriate architecture is crucial for effective processing. A client-server architecture model will be adopted to optimize the performance of limited user devices by offloading heavy processing tasks to the server, making client devices primarily an interface for users. The system architecture is illustrated in the figure below.

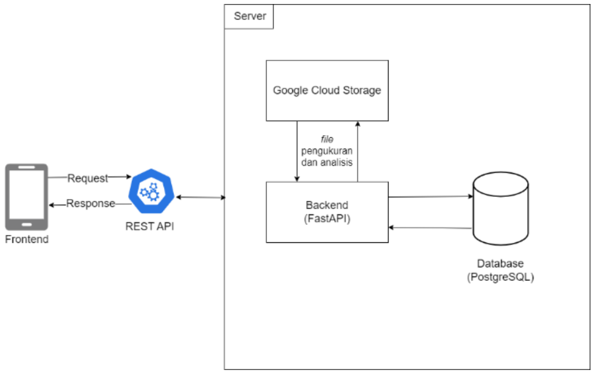

Fig. 10. System architecture

In this project, the server for the data measurement analysis system is divided into two main components: the backend and the database. Communication between the client and server is handled via a REST API, responsible for the application's activities. The backend system connects to a PostgreSQL database to access and store data. Additionally, the backend uses Google Cloud Storage to handle requests related to measurement data, storing both the raw voltammetry measurement data files and the analyzed data files. The analysis results are designed to be stored in Google Cloud Storage, allowing users to review their measurement data analysis without repeatedly pressing the analysis button, thereby minimizing computation costs. This ensures the system is efficient and resource effective.

3.3. Frontend System

3.3.1. Analysis Page

In this page, users can upload their data file into the system. Users need to input the name of the measurement and the description if needed. For CV data types, it only needs one file. But, for DPV

data types, it needs two file. So, the frontend system will check, if the amount of file that each of types is correct, then the upload button is clickable. Client also will check the type of file, because it needs to csv file.

After the users upload and click the upload, frontend will send the data first to server. First, client will send data to server and the server will check is the file is valid or not. After check if file is valid, server will store file to cloud storage and notify the client if the storing is success or not.

If the storing process is success, client will send request again to server to analyze the file. After the analysis is finish, server will send back the result to frontend and frontend will display the result to users.

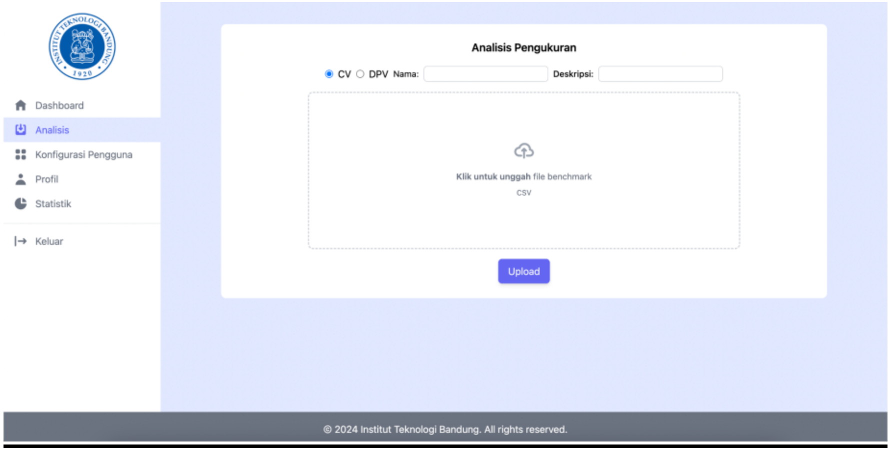

Fig. 11. Analysis page

3.3.2. History Page

In history page, users can see their history of analysis. They can also delete the data. In client, there will be some checker. If the users is admin or laborant, they can also see the detail of analysis, which is chart page. But, if they are not, they only can see the result, which is potential they have heart disease.

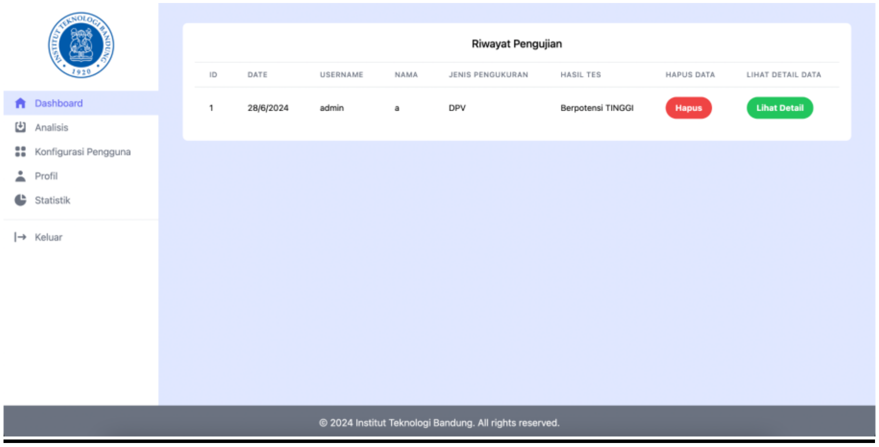

Fig. 12. History page

3.3.3. Chart Page

For the chart system, we will compare between 4 open source libraries to see which library is most suitable for this case. The four libraries are Recharts, Chart Js, Nivo, and Plotly. Because there are 2 types of data that will be displayed (CV and DPV), the result will be created in a table to make it easier to read.

3.3.3.1. Chart Testing

Testing for charts will be conducted using one data file each for CV and DPV types. The data used to test CV is CV Ascorbid Acid - 2.csv, while the data for testing DPV is DPV Ascorbic Acid - 1.csv.

The testing will involve displaying each chart for CV and DPV on the website using the four previously defined frameworks. The testing environment will be on a blank webpage that only contains chart displays to minimize distractions from external influences.

Additionally, testing will be conducted by comparing the output charts with benchmark charts obtained from the PSTrace software. The comparison aims to determine whether the output from the final project software is accurate or more accurate compared to the benchmark software.

The testing will use the factors of chart completeness based on the material in section 2.3.4.1. These factors are:

1. Annotation
2. Interactivity
3. Manipulating Parameters

Annotation will check whether the chart allows for the addition of reference lines. The purpose of the reference lines is to assist the user in seeing the difference between the peak and baseline values. This value is useful for determining whether an individual is at high or low risk of heart disease.

Interactivity will check whether a text box containing an explanation of the actual values appears when a user hovers over the chart line. This check aims to help users avoid misinterpreting data.

Manipulating Parameters will check whether users can filter a line. For example, when two lines are displayed, filtering can be applied to show only one line. This check aims to help users simplify complex charts for easier interpretation.

Table. 5. Table of DPV Data Chart Completeness Test Results.

No	Factors	Recharts	Chart Js	Nivo	Plotly	PSTrace
1.	Annotation - reference line	Yes	No	No	Yes	No
2.	Interactivity - details	Yes	No	Yes	Yes	No
3.	Interactivity - manipulating parameters	No	No	No	Yes	No

Based on table 5 that compared Recharts, Chart Js, Nivo, Plotly, and PSTrace, we can see that Plotly is very suitable with our need to analyze data. Plotly has advantages in annotation, especially in reference line, and interactivity, with factors in details and manipulating parameters. Compared with control software, Plotly is very distinguished in these factors, and this concludes that we will use Plotly as a software framework for this software.

3.3.3.2. Result CV

Table. 3. Result Frontend for CV data types

<table>
<tr><td>Image</td><td>Library</td></tr>
<tr><td>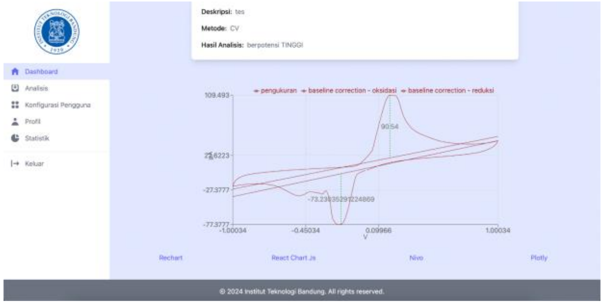
</td><td>Recharts</td></tr>
<tr><td>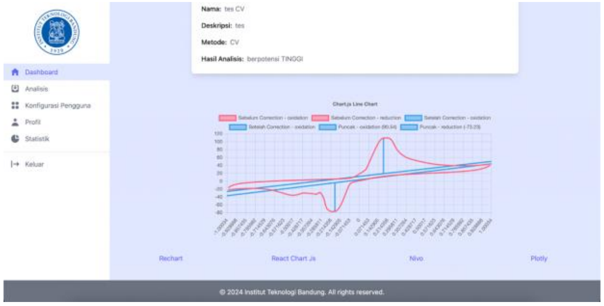
</td><td>Chart Js</td></tr>
<tr><td>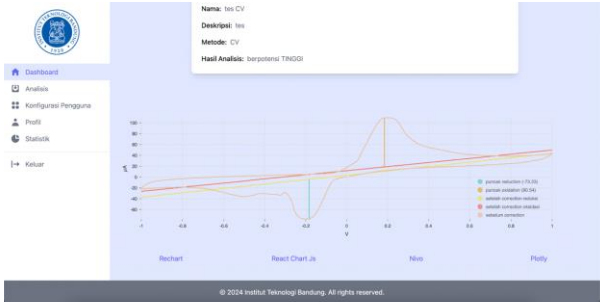
</td><td>Nivo</td></tr>
</table>

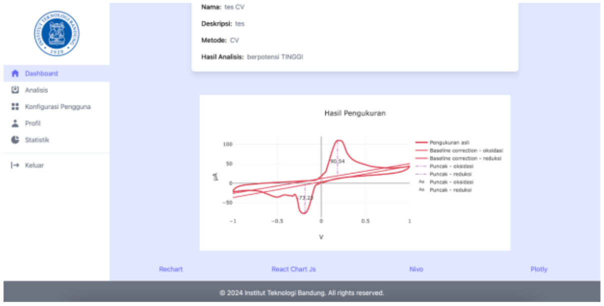	Plotly

3.3.3.3. Result DPV

Table. 4. Result Frontend for DPV data types.

Image	Library
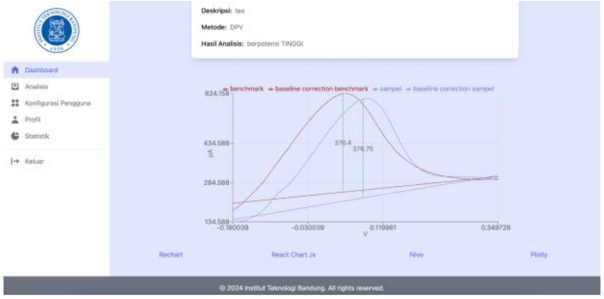	Recharts
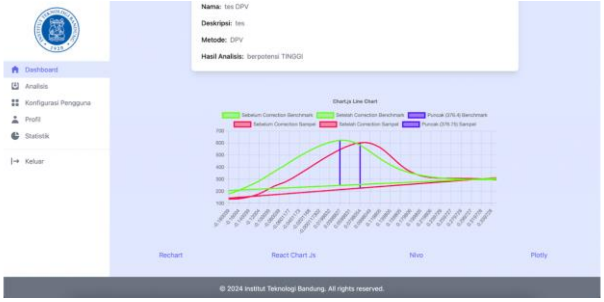	Chart Js

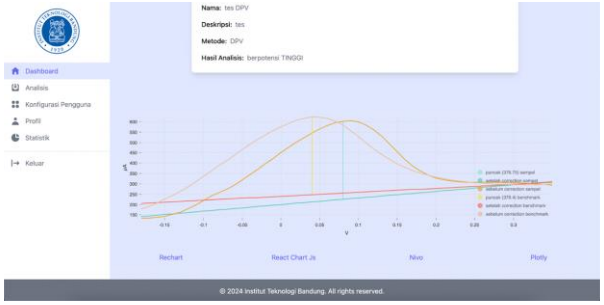	Nivo
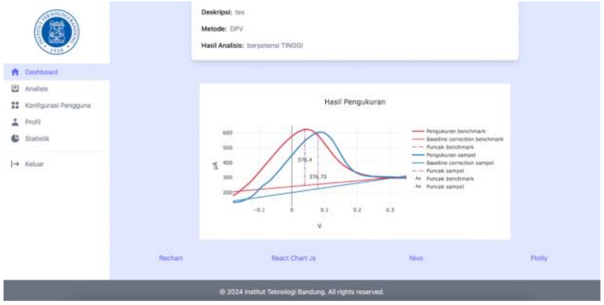	Plotly

3.4. Testing for Data Analysis

3.4.1. Load Testing

Load testing aims to assess the speed, response time, reliability, and scalability of the system. This method identifies weak points and measures system performance under the load generated by multiple concurrent users. Locust is the tool used for this load testing.

The load testing focused on several key application features that are frequently accessed:

- Retrieving the list of measurements.
- Opening the measurement detail page.

The load testing was performed by simulating a number of concurrent users making requests to the server for these key features. The configuration for the load testing is as follows:

- Concurrent Users: 100 users, assuming the application is used by specific users such as lab technicians and some general public users as an initial test case.
- Testing Duration: 90 seconds, based on the average time users spend on a website, which is approximately 90 seconds.

Request	Parameter	Value
GET /api/measurements	Total number of requests	8050
	Average response time	330ms

	Success rate	100%
	Maximum response time	1280ms
GET /api/measurements/{id}	Total number of requests	8024
	Average response time	410ms
	Success rate	100%
	Maximum response time	1314ms

The load testing on the backend system demonstrated high throughput and consistent performance, with a total of 16,074 requests processed in 90 seconds. The average response times were 330ms for retrieving the list of measurements and 410ms for opening measurement detail pages, both maintaining a 100% success rate. The maximum response times were 1280ms and 1314ms, respectively, indicating the system's capability to handle peak loads without significant performance degradation. These results confirm that the backend system is robust, reliable, and scalable, efficiently supporting concurrent user requests.

4. Discussion

4.1. Data Visualization

The comparison between the implemented system and PSTrace software shows that, in the CV method, the implemented system's curve has multiple peaks with varying baselines, while PSTrace provides sharper, clearer oxidation and reduction peaks with a more stable baseline. In the DPV method, both systems show a single prominent peak with a corrected baseline, indicating high sensitivity. This demonstrates that key features such as peaks and baselines can be identified in the implemented system, and PSTrace can be used as a tool for comparison, offering more precise and visually clearer results.

Graphical Visualization Monitoring System Built	PSTrace

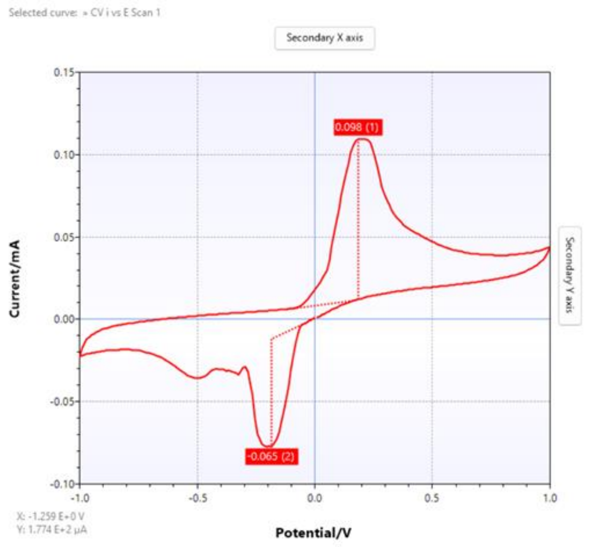

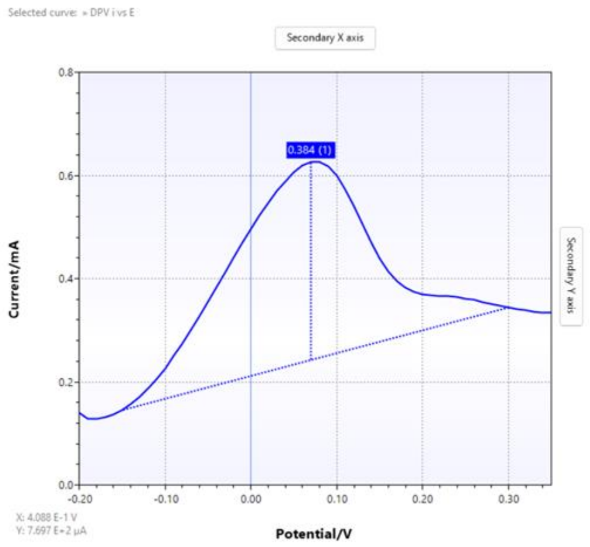

4.2. Chart

Based on table 5 from 3.3.3.3., we can see that Plotly has anything that the software needs based on material 2.3.4.1. Plotly is the most suitable library for this case if we compare it to the benchmark software, PSTrace. Also, Plotly has more advantages compared with the other libraries like Recharts, Chart Js, and Nivo. So, it can be concluded that for this software, we will use Plotly as a chart library.

The discussion section should explore the significance and implications of the findings and their limitations, with reference to all other relevant studies and future research perspectives. The discussion section should not be a repetition of the results section.

5. Conclusions

The study successfully developed a web-based system that processes raw voltammetry data into interpretable results for heart disease detection. By integrating electrochemical techniques, cloud-based storage, and graphical visualization, the system provides accurate predictions of cardiac biomarker concentrations, enhancing the ability to detect heart disease early. The backend system, built using FastAPI and PostgreSQL, demonstrated scalability and efficiency in handling large datasets, while the frontend, leveraging React and Plotly, offered an interactive and user-friendly experience.

This research addresses a significant gap in translating raw voltammetry data into actionable insights, making early detection of heart disease more accessible and reliable. By automating data analysis and visualization, the system reduces manual errors, facilitates remote monitoring, and empowers healthcare professionals to make timely and informed diagnoses. The findings underscore the importance of combining advanced data processing and visualization technologies to tackle critical healthcare challenges.

Funding:

This work was supported by School of Electrical Engineering and Informatics Institut Teknologi Bandung under P2MI program (Number 753/IT1.C12/TU.10/2024, February 12th, 2024).

References:

1. World Health Organization. Cardiovascular diseases (CVDs). Accessed July 6, 2024. [Online]. Available: https://www.who.int/news-room/factsheets/detail/cardiovascular-diseases-(cvds)
2. Spinner, N. What is a potentiostat and how does it work? Pine Research; 2023.
3. Anshori, I., Mufiddin, G. F., Ramadhan, I. F., Ariasena, E., Harimurti, S., Yunkins, H., & Kurniawan, C. Design of smartphone-controlled low-cost potentiostat for cyclic voltammetry analysis based on ESP32 microcontroller. Sensing and Bio-Sensing Research. 2022; 36:100490. doi:10.1016/j.sbsr.2022.100490.
4. Nicholson, R. S. Theory and application of cyclic voltammetry for measurement of electrode reaction kinetics. Anal. Chem. 1965; 37(11): 1351–1355. doi:10.1021/ac60230a016.
5. Elgrishi, N., Rountree, K. J., McCarthy, B. D., Rountree, E. S., Eisenhart, T. T., & Dempsey, J. L. A practical beginner's guide to cyclic voltammetry. J. Chem. Educ. 2018; 95(2): 197–206. doi:10.1021/acs.jchemed.7b00361.
6. Peroff, A. Differential pulse voltammetry (DPV). Pine Research.
7. Shen, Q., Liu, M., Lü, Y., & Zhang, D. Label-free electrochemical immunosensor based on a functionalized ionic liquid and helical carbon nanotubes for the determination of cardiac troponin I. ACS Omega. 2019; 4(7): 11888-11892. doi:10.1021/acsomega.9b01152.

8. Kirk, A. Data visualization: A successful design process. Birmingham: Packt Publishing; 2012.
