# Supplementary material for: Algorithm-assisted interpretation of cyclic and differential pulse voltammetry for cardiac troponin detection
Source: PLoS One. 2026 May 13;21(5):e0348348. doi: 10.1371/journal.pone.0348348 (PMC13170963; doi:10.1371/journal.pone.0348348)
Supplement: Attachment — Submitted filename: Response to Reviewers.docx [file pone.0348348.s003.docx]

**Journal Requirements:**

When submitting your revision, we need you to address these additional requirements.

1.Please ensure that your manuscript meets PLOS ONE's style requirements, including those for file naming. The PLOS ONE style templates can be found at

<https://journals.plos.org/plosone/s/file?id=wjVg/PLOSOne_formatting_sample_main_body.pdf> and

[https://journals.plos.org/plosone/s/file?id=ba62/PLOSOne_formatting_sample_title_authors_affiliations.pdf](https://track.editorialmanager.com/CL0/https:%2F%2Fjournals.plos.org%2Fplosone%2Fs%2Ffile%3Fid=ba62%2FPLOSOne_formatting_sample_title_authors_affiliations.pdf/1/010f019481bf7efc-c760d5a2-9232-488b-862a-14f9bdd943bb-000000/54GwEHuLBT_S6A9QQYZ8jpqcOdfnHsQt4MtxeigYRhs=194)

**Response:**

We have revised the manuscript to conform to the PLOS ONE style requirements, including formatting and file naming, as outlined in the provided templates.

2. Please note that funding information should not appear in any section or other areas of your manuscript. We will only publish funding information present in the Funding Statement section of the online submission form. Please remove any funding-related text from the manuscript.

**Response:**

Thank you for the clarification. We have removed the funding-related text from the manuscript as requested. The funding information is now provided solely in the Funding Statement section of the online submission form.

3. We note that the grant information you provided in the ‘Funding Information’ and ‘Financial Disclosure’ sections do not match.

When you resubmit, please ensure that you provide the correct grant numbers for the awards you received for your study in the ‘Funding Information’ section.

**Response:**

Thank you for bringing this to our attention. We have reviewed and corrected the grant information to ensure consistency between the 'Funding Information' and 'Financial Disclosure' sections. The correct grant number has now been entered in the ‘Funding Information’ section of the online submission form.

4. Thank you for stating the following financial disclosure:

“The funding is from P2MI 2024 Program of School of Electrical Engineering and Informatics Institut Teknologi Bandung”

Please state what role the funders took in the study. If the funders had no role, please state: "The funders had no role in study design, data collection and analysis, decision to publish, or preparation of the manuscript."

If this statement is not correct you must amend it as needed.

Please include this amended Role of Funder statement in your cover letter; we will change the online submission form on your behalf.

**Response:**
Thank you for the clarification. We confirm that the funders had no involvement in the study. The following statement has been added to the cover letter as requested:
**"The funders had no role in study design, data collection and analysis, decision to publish, or preparation of the manuscript."**

5. When completing the data availability statement of the submission form, you indicated that you will make your data available on acceptance. We strongly recommend all authors decide on a data sharing plan before acceptance, as the process can be lengthy and hold up publication timelines. Please note that, though access restrictions are acceptable now, your entire data will need to be made freely accessible if your manuscript is accepted for publication. This policy applies to all data except where public deposition would breach compliance with the protocol approved by your research ethics board. If you are unable to adhere to our open data policy, please kindly revise your statement to explain your reasoning and we will seek the editor's input on an exemption. Please be assured that, once you have provided your new statement, the assessment of your exemption will not hold up the peer review process.

**Response:**

Thank you for the important reminder. The link to the repository is https://drive.google.com/drive/folders/1XJ-pjEaOa-UFj499yzcPW7GTk--q6ilt?usp=sharing

We fully support PLOS ONE’s open data policy and will ensure compliance before final publication.

6. We note that Figure 11 and 12 in your submission contain copyrighted images. All PLOS content is published under the Creative Commons Attribution License (CC BY 4.0), which means that the manuscript, images, and Supporting Information files will be freely available online, and any third party is permitted to access, download, copy, distribute, and use these materials in any way, even commercially, with proper attribution. For more information, see our copyright guidelines: http://journals.plos.org/plosone/s/licenses-and-copyright.

We require you to either (a) present written permission from the copyright holder to publish these figures specifically under the CC BY 4.0 license, or (b) remove the figures from your submission:

a. You may seek permission from the original copyright holder of Figure 11 and 12 to publish the content specifically under the CC BY 4.0 license.

We recommend that you contact the original copyright holder with the Content Permission Form (http://journals.plos.org/plosone/s/file?id=7c09/content-permission-form.pdf) and the following text:

“I request permission for the open-access journal PLOS ONE to publish XXX under the Creative Commons Attribution License (CCAL) CC BY 4.0 (http://creativecommons.org/licenses/by/4.0/). Please be aware that this license allows unrestricted use and distribution, even commercially, by third parties. Please reply and provide explicit written permission to publish XXX under a CC BY license and complete the attached form.”

Please upload the completed Content Permission Form or other proof of granted permissions as an "Other" file with your submission.

In the figure caption of the copyrighted figure, please include the following text: “Reprinted from [ref] under a CC BY license, with permission from [name of publisher], original copyright [original copyright year].”

b. If you are unable to obtain permission from the original copyright holder to publish these figures under the CC BY 4.0 license or if the copyright holder’s requirements are incompatible with the CC BY 4.0 license, please either i) remove the figure or ii) supply a replacement figure that complies with the CC BY 4.0 license. Please check copyright information on all replacement figures and update the figure caption with source information. If applicable, please specify in the figure caption text when a figure is similar but not identical to the original image and is therefore for illustrative purposes only.

**Response:**

Thank you for bringing this to our attention. We have reviewed Figures 11 and 12 and acknowledge that they include copyrighted content. In response, we have taken the following action:

We have removed Figures 11 and 12 from the revised manuscript to comply with the CC BY 4.0 license requirements. These figures have been replaced with original illustrations that we created ourselves for the purpose of this publication.

7. We note you have included a table to which you do not refer in the text of your manuscript. Please ensure that you refer to Table 1-4 in your text; if accepted, production will need this reference to link the reader to the Table.

**Response:**

Thank you for pointing this out. We have revised the manuscript to ensure that Tables 1 through 4 are explicitly referenced in the main text at appropriate locations. These references now guide the reader to the relevant data and support the corresponding discussions.

**Comments to the Author**

1. Is the manuscript technically sound, and do the data support the conclusions?

The manuscript must describe a technically sound piece of scientific research with data that supports the conclusions. Experiments must have been conducted rigorously, with appropriate controls, replication, and sample sizes. The conclusions must be drawn appropriately based on the data presented.

Reviewer #1: Yes

Reviewer #2: Partly

**Response:**

We appreciate the reviewers’ evaluations. In response to Reviewer #2's note, we have reviewed and revised relevant sections of the manuscript to strengthen the connection between the data and the conclusions. We have clarified the explanation of the algorithms used for data analysis, added further justification for using DPV over CV in the context of sensitivity, and elaborated on how the backend system ensures reliable prediction outcomes. These improvements are reflected particularly in the revised **Results & Discussion** section (Section 3), as well as the **Conclusion**.

2. Has the statistical analysis been performed appropriately and rigorously?

Reviewer #1: Yes

Reviewer #2: N/A

**Response**:

We thank the reviewer for the positive assessment. We confirm that the data analysis procedures, including peak and baseline identification as well as threshold-based decision-making, were conducted rigorously following standard practices in electrochemical signal processing. No additional statistical testing was required beyond these signal analysis methods.

3. Have the authors made all data underlying the findings in their manuscript fully available?

The PLOS Data policy requires authors to make all data underlying the findings described in their manuscript fully available without restriction, with rare exception (please refer to the Data Availability Statement in the manuscript PDF file). The data should be provided as part of the manuscript or its supporting information or deposited to a public repository. For example, in addition to summary statistics, the data points behind means, medians and variance measures should be available. If there are restrictions on publicly sharing data—e.g., participant privacy or use of data from a third party—those must be specified.

Reviewer #1: Yes

Reviewer #2: Yes

**Response:**

We appreciate the reviewers’ confirmation. We have ensured that all relevant data underlying our findings—including raw voltammetry measurement files, analysis outputs, and visualizations—are made fully available as part of the manuscript's supplementary information and have also been deposited in a public repository as noted in the Data Availability Statement.

The research data is available in <https://drive.google.com/drive/folders/1XJ-pjEaOa-UFj499yzcPW7GTk--q6ilt?usp=sharing>

4. Is the manuscript presented in an intelligible fashion and written in standard English?

PLOS ONE does not copyedit accepted manuscripts, so the language in submitted articles must be clear, correct, and unambiguous. Any typographical or grammatical errors should be corrected at revision, so please note any specific errors here.

Reviewer #1: Yes

Reviewer #2: Yes

**Response:**

We thank the reviewers for their positive feedback. We have carefully proofread the manuscript to ensure clarity, correctness, and consistency in standard English. Minor typographical and grammatical issues, where identified, have been corrected in this revision.

5. Review Comments to the Author

Please use the space provided to explain your answers to the questions above. You may also include additional comments for the author, including concerns about dual publication, research ethics, or publication ethics. (Please upload your review as an attachment if it exceeds 20,000 characters)

Reviewer #1: The manuscript presents a comprehensive study on the development of an interactive graphical visualization system for heart disease detection using electrochemical voltammetry techniques, specifically Differential Pulse Voltammetry (DPV) and Cyclic Voltammetry (CV). Overall, the paper is well organized. Therefore, I would like to recommend the publication of this work in PLOS ONE after a minor revision. The details are as follows:

**Comment 1:** Introduction: Suggest to a more detailed discussion of existing solutions and their limitations.

**Response:**

Thank you for the constructive suggestion. In response, we have expanded the Introduction section to include a more detailed discussion of existing solutions for heart disease detection using electrochemical techniques and their limitations. Specifically, we have highlighted the challenges in interpreting raw voltammetry data manually, the lack of user-friendly interfaces in current systems, and the limitations in real-time data accessibility and automated analysis. These revisions help to better contextualize the motivation and contribution of our proposed system.

**Comment 2:** The description of the sensor reader and data analysis process is thorough, but more technical details on the algorithms used for data processing would be beneficial.

**Response:**

Thank you for the insightful feedback. In response, we have added more technical details regarding the algorithms used for baseline correction and peak detection in Section 3.2 of the manuscript. Specifically, we elaborated on the use of polynomial fitting for cyclic voltammetry (CV) and asymmetric least squares (ALS) for differential pulse voltammetry (DPV), including the rationale for choosing these methods and how they handle signal noise and baseline drift. We also clarified how these algorithms integrate with the backend system to produce reliable current response measurements for diagnostic purposes.

**Comment 3:** Raw data screenshots do not add much value or insight. Suggest removing these figures if no additional insight can be provided using them.

**Response:**
We appreciate the reviewer’s observation. In response, we have removed the raw data screenshots (Figures 3 and 4) from the revised manuscript to maintain focus and clarity. Additionally, we have ensured that the key characteristics of the raw data are now clearly described in the text to preserve the necessary context without relying on those visual elements.

**Comment 4**: The interpretation of the CV data and correlation to heart disease detection is unclear. Please add more details.

**Response:**
Thank you for the valuable feedback. In the revised manuscript, we have clarified the role of cyclic voltammetry (CV) in the context of our system. Although DPV is the primary method used for heart disease detection due to its higher sensitivity, we included CV to support system functionality testing and sensor behaviour analysis. CV helps identify redox behaviour and validate the presence of electrochemical reactions, which supports calibration and comparison. However, the final diagnostic decision regarding heart disease is based on DPV data, as CV lacks sufficient resolution for reliable quantitative assessment of cardiac troponin levels. These clarifications have been added to Section 3.2.1 and the Discussion section.

**Reviewer #2:** Manuscript: PONE-D-24-56032 entitled “Interactive Graphical Visualization for Electrochemical Voltammetry Techniques of Differential Pulse Voltammetry and Cyclic Voltammetry”

This study provided a thorough understanding of the rapid identification of crucial biomarkers, such as cardiac troponin, using electrochemical voltammetry techniques. The electrochemical testing data revealed reliability and effectiveness in developing these biomarkers, as the author(s) claimed in this study. The authors tried to explain their works scientifically. However, the following quarries cleared before acceptance for publication of this manuscript in your esteemed journal.

1. In the abstract section, what new findings (results) are reported by the authors from this study? What novelty of study? It should elaborate.

**Response:**

Thank you for highlighting this important point. In response, we have revised the abstract to clearly emphasize the novelty and key findings of our study. Specifically, the novelty lies in the integration of an automated backend system using FastAPI and cloud-based storage that processes raw voltammetry data (CV and DPV) and converts it into diagnostic predictions via a user-friendly web interface. Additionally, we highlight the implementation of specific algorithms for baseline correction (polynomial fit for CV and ALS for DPV) and their role in accurate cardiac troponin detection.

The revised abstract now explicitly states:

“A key novelty of this research lies in the integration of automated baseline correction and peak detection algorithms—specifically polynomial fitting for cyclic voltammetry (CV) and asymmetric least squares (ALS) for differential pulse voltammetry (DPV)—within a user-friendly interface accessible even to non-expert users.”

These updates clarify both the scientific contribution and the practical innovation of the study.

2. The introduction section lacks sufficient references, with only a few cited. Are there no previous studies on this topic or related fields? Clarify the rationale, objectives, and novelty of this study.

**Response:**

Thank you for your valuable comment. In response, we have revised the Introduction section to include additional references to prior studies relevant to electrochemical voltammetry for cardiac biomarker detection, particularly those focusing on cardiac troponin and biosensor-based approaches. These citations provide better context and demonstrate the existing research landscape.

Furthermore, we have clarified the rationale of this study, which stems from the difficulty in interpreting raw voltammetry data without specialized tools or expertise. We emphasized the objective, which is to develop a web-based system that automates data analysis and visualization for early heart disease detection. We also highlighted the novelty, which lies in:

- the integration of CV and DPV data processing using tailored baseline correction algorithms,
- the implementation of a cloud-connected, real-time analysis system,
- and its accessibility for both clinical and non-specialist users.

These improvements have been incorporated into the revised introduction for greater clarity and scientific grounding.

3. In the Materials & Methods section, ….. oxidation reaction occurs on the auxiliary electrode…. Was the electrochemical sensor a three-electrode system or a two-electrode system? It should be a three-electrode system (as the authors mentioned, used auxiliary electrodes). If so, what was the reference electrode?

**Response:**

We appreciate the reviewer’s attention to this important clarification. The electrochemical sensor used in this study is indeed a three-electrode system, as correctly noted. It consists of:

- **Working Electrode (WE)** – where the redox reaction (oxidation/reduction) of the analyte (cardiac troponin) occurs,
- **Reference Electrode (RE)** – which provides a stable reference potential (Ag/AgCl was used), and
- **Counter (Auxiliary) Electrode (CE)** – which completes the circuit and balances the current flow.

We have revised the relevant section in Materials & Methods to correct the statement and clearly state that the redox reaction primarily occurs on the working electrode, not the auxiliary electrode. The role of each electrode has also been further clarified in the revised manuscript to avoid confusion.

4. This section lacks clarity and requires revision with proper references. Shorten the description of CV and DPV techniques, which are well-defined.

**Response:**

Thank you for your helpful suggestion. In response, we have revised the Materials & Methods section to improve clarity and conciseness. The descriptions of cyclic voltammetry (CV) and differential pulse voltammetry (DPV) have been shortened to focus only on the essential principles relevant to this study. As these techniques are well-established, we have also added appropriate references to support the brief descriptions, rather than providing lengthy explanations.

These revisions ensure the section remains scientifically accurate while maintaining focus on the implementation and context of the methods in our system.

5. In the Results & Discussion section, why did the author(s) not describe the results of the CV and DPV graphs? Additionally, are the data presented in the CV and DPV raw? This reviewer believes these electrochemical techniques offer quantitative information, which should not be as raw data.

**Response:**

Thank you for the valuable comment. In the revised Results & Discussion section, we have now included a more detailed explanation and interpretation of the CV and DPV data.

We clarified that the CV data was primarily used to evaluate redox behaviour and validate sensor functionality, rather than for direct quantification. Meanwhile, the DPV data was used for quantitative analysis due to its higher sensitivity and better signal resolution. We also explained how the raw voltammetry data (current vs. potential) undergoes processing—specifically, baseline correction and peak detection—before being used to extract quantitative metrics such as current response values, which are then used for heart disease risk prediction.

Furthermore, we emphasized that although the figures initially showed raw formats, the backend system processes these data into analysed results, which form the basis for clinical interpretation. We have now made this data flow and transformation more explicit in the revised manuscript to avoid misunderstanding.

6. Also, the discussion section is poor and should be elaborated with proper citations.

**Response:**

Thank you for this important observation. We have revised and expanded the Discussion section to provide a deeper analysis of the results and their implications. Specifically, we are now:

- Elaborate on the effectiveness of the DPV method for detecting cardiac troponin, supported by recent literature,
- Compare our system's performance with existing approaches in terms of data visualization, automation, and accessibility,
- Discuss the advantages and limitations of the implemented algorithms (ALS and polynomial fitting),
- Highlight the practical significance of integrating cloud-based analysis and user-friendly interfaces for broader healthcare applications.

We have also added several new references to support these points and place our findings within the context of current research in electrochemical biosensing and digital health technology.

7. Needed to correct some grammatical errors as indicated in revised manuscript.

**Response:**

Thank you for pointing this out. We have carefully reviewed the manuscript and corrected the grammatical errors as indicated. We also conducted an additional round of proofreading to improve the overall clarity, consistency, and readability of the text to meet the standard of formal scientific English.
