# Supplementary material for: Algorithm-assisted interpretation of cyclic and differential pulse voltammetry for cardiac troponin detection
Source: PLoS One. 2026 May 13;21(5):e0348348. doi: 10.1371/journal.pone.0348348 (PMC13170963; doi:10.1371/journal.pone.0348348)
Supplement: Attachment — Submitted filename: Response_to_Reviewers_auresp_2.docx [file pone.0348348.s004.docx]

**Response to Reviewers**

**Comments to the Author**

1. If the authors have adequately addressed your comments raised in a previous round of review and you feel that this manuscript is now acceptable for publication, you may indicate that here to bypass the “Comments to the Author” section, enter your conflict of interest statement in the “Confidential to Editor” section, and submit your "Accept" recommendation.

Reviewer #2: All comments have been addressed

Reviewer #3: All comments have been addressed

**Response**

We sincerely thank Reviewer #2 and Reviewer #3 for confirming that all comments have been adequately addressed. In this revision, we have additionally improved consistency in citation style and refined the manuscript for clarity and readability as part of editorial polishing.

2. Is the manuscript technically sound, and do the data support the conclusions?

The manuscript must describe a technically sound piece of scientific research with data that supports the conclusions. Experiments must have been conducted rigorously, with appropriate controls, replication, and sample sizes. The conclusions must be drawn appropriately based on the data presented.

Reviewer #2: Yes

Reviewer #3: Partly

**Response**

We thank the reviewers for their assessment. In response to Reviewer #3’s comment, we have further clarified the methodological description and strengthened the linkage between the presented data and the conclusions. Specifically, we refined the explanation of the experimental setup, data processing workflow, and interpretation of the CV and DPV results, emphasizing that the conclusions are directly derived from the analyzed data. These revisions improve clarity and technical transparency without altering the underlying results or conclusions of the study.

3. Has the statistical analysis been performed appropriately and rigorously?

Reviewer #2: Yes

Reviewer #3: No

**Response**

We thank the reviewers for their evaluation. In this study, the data analysis is based on established electrochemical signal processing methods rather than inferential statistical testing. We have clarified this distinction in the revised manuscript by explicitly describing the analytical procedures used for baseline correction, peak detection, and signal interpretation for CV and DPV data. These methods were applied consistently across all measurements and provide an appropriate and rigorous basis for the conclusions drawn

4. Have the authors made all data underlying the findings in their manuscript fully available?

The [PLOS Data policy](http://www.plosone.org/static/policies.action#sharing) requires authors to make all data underlying the findings described in their manuscript fully available without restriction, with rare exception (please refer to the Data Availability Statement in the manuscript PDF file). The data should be provided as part of the manuscript or its supporting information, or deposited to a public repository. For example, in addition to summary statistics, the data points behind means, medians and variance measures should be available. If there are restrictions on publicly sharing data—e.g. participant privacy or use of data from a third party—those must be specified.

Reviewer #2: Yes

Reviewer #3: Yes

**Response**

We thank the reviewers for their confirmation. All data underlying the findings described in the manuscript, including raw voltammetry measurement files and processed analysis outputs, are fully available through the repository indicated in the Data Availability Statement.

5. Is the manuscript presented in an intelligible fashion and written in standard English?

PLOS ONE does not copyedit accepted manuscripts, so the language in submitted articles must be clear, correct, and unambiguous. Any typographical or grammatical errors should be corrected at revision, so please note any specific errors here.

Reviewer #2: Yes

Reviewer #3: No

**Response**

We thank the reviewers for their feedback. In response to Reviewer #3’s comment, we have carefully revised and polished the manuscript to improve clarity, consistency, and readability in standard scientific English. Typographical and grammatical errors have been corrected, and several sentences have been rephrased to enhance precision and flow, without changing the scientific content of the manuscript.

6. Review Comments to the Author

Please use the space provided to explain your answers to the questions above. You may also include additional comments for the author, including concerns about dual publication, research ethics, or publication ethics. (Please upload your review as an attachment if it exceeds 20,000 characters)

Reviewer #2: I read the revised manuscript and found that it addresses previous comments well and incorporates the revisions. I, however, am not sure whether the style of citing the reference in the text is ..... [1]. or ...... . [1]. Both styles are used in the manuscript; it should be corrected.

Reviewer #3: English of manuscript is below the required standards and needs a careful polish.
Section Potentiostat and voltammetry needs a careful revision. Why SPCE electrode is used? Ag/AgCl paste!!!
what is the reference electrode? non-useful parts eg. definitions needs to be removed.
the title of Anshori paper in page 5 must be deleted. what is the baseline correction system? Conclusion section must be re-prepared as more informative. Statistical analysis section or methods?

**Response**

We thank the reviewers for their constructive comments.

In response to Reviewer #2, we have standardized the in-text citation format throughout the manuscript to ensure consistency with the journal’s reference style.

In response to Reviewer #3, we have carefully polished the English language to meet the journal’s standards. The Potentiostat and Voltammetry section has been revised to improve clarity, explicitly justify the use of the SPCE, and clearly describe the reference electrode configuration. Non-essential definitions have been removed, and the title of the cited work by Anshori has been deleted from the main text. We have also clarified the baseline correction workflow, revised the Conclusion section to be more informative, and explicitly positioned the analytical procedures within the Methods section to address concerns regarding statistical analysis. These revisions improve clarity and presentation without altering the scientific content of the manuscript.

7. PLOS authors have the option to publish the peer review history of their article ([what does this mean?](https://journals.plos.org/plosone/s/editorial-and-peer-review-process#loc-peer-review-history)). If published, this will include your full peer review and any attached files.


If you choose “no”, your identity will remain anonymous but your review may still be made public.


**Do you want your identity to be public for this peer review?** For information about this choice, including consent withdrawal, please see our [Privacy Policy](https://www.plos.org/privacy-policy).

Reviewer #2: **Yes:** Jagadeesh Bhattarai

Reviewer #3: No

**Response**

We acknowledge the reviewers’ choices regarding the publication of peer review history and comply with the journal’s policy on peer review transparency and anonymity.
