# Supplementary material for: Algorithm-assisted interpretation of cyclic and differential pulse voltammetry for cardiac troponin detection
Source: PLoS One. 2026 May 13;21(5):e0348348. doi: 10.1371/journal.pone.0348348 (PMC13170963; doi:10.1371/journal.pone.0348348)
Supplement: Attachment — Submitted filename: Response_to_Reviewers_auresp_3.docx [file pone.0348348.s005.docx]

**Response to Reviewers**

**Journal Requirements:**

1. If the reviewer comments include a recommendation to cite specific previously published works, please review and evaluate these publications to determine whether they are relevant and should be cited. There is no requirement to cite these works unless the editor has indicated otherwise.

**Response:**

We appreciate the reviewer’s suggestion regarding the inclusion of additional references. All recommended publications have been carefully reviewed and evaluated for their relevance to the scope and methodology of this study. Relevant works have been incorporated into the revised manuscript where appropriate to strengthen the theoretical background and support the proposed approach. For suggested references that were not directly aligned with the focus of this study, we have opted not to include them to maintain the clarity and specificity of the manuscript.

2. Please review your reference list to ensure that it is complete and correct. If you have cited papers that have been retracted, please include the rationale for doing so in the manuscript text, or remove these references and replace them with relevant current references. Any changes to the reference list should be mentioned in the rebuttal letter that accompanies your revised manuscript. If you need to cite a retracted article, indicate the article’s retracted status in the References list and also include a citation and full reference for the retraction notice.

**Response:**

We thank the reviewer for this important comment. The reference list has been carefully reviewed to ensure completeness, accuracy, and relevance. All cited works were rechecked to confirm their validity, and no retracted articles were identified in the revised manuscript.

In addition, minor updates have been made to improve the quality of the references, including verification of bibliographic details and replacement of outdated or less relevant sources with more recent and appropriate publications where necessary. These revisions have strengthened the scientific grounding of the manuscript.

All changes to the reference list have been reflected in the revised manuscript and are documented accordingly in this response.

**Additional Editor Comments:**

- Title of manuscript must be informative and reflect the content of the work closely.

**Response:**

We thank the editor for this valuable suggestion. The manuscript title has been revised to more clearly reflect the scope, methodology, and contribution of the study. The updated title emphasizes the algorithm-assisted interpretation of voltammetry data, the integration of cyclic and differential pulse voltammetry techniques, and its application to cardiac troponin analysis. This revision improves clarity, specificity, and alignment with the content of the manuscript.

- English of manuscript needs to be improved carefully. The abstract must be prepared as more informative. For example, "The potential for heart disease can be detected using a sensor reader that utilizes electrochemical voltammetry techniques, enabling the rapid identification of critical biomarkers such as cardiac troponin." must be improved as "The risk of heart disease can be identified through a sensor that employs electrochemical voltammetry, a technique that allows for the rapid and precise detection of critical biomarkers, including cardiac troponin." etc...

Please Improve the text carefully and submit the revised version.

**Response:**

We thank the editor for this valuable and constructive comment. The manuscript has been carefully revised to improve the overall quality, clarity, and readability of the English throughout the text. Grammatical structure, sentence flow, word choice, and academic style have been thoroughly refined to ensure that the manuscript is written in clear and standard scientific English.

In particular, the abstract has been substantially revised to make it more informative and aligned with the scope, methodology, and main findings of the study. The revised abstract now more clearly describes: (1) the research background and problem addressed, (2) the algorithm-assisted approach used for cyclic voltammetry (CV) and differential pulse voltammetry (DPV) analysis, (3) the main signal-processing methods applied, and (4) the principal findings and contribution of the study. In addition, several sentences across the Introduction, Results and Discussion, and Conclusion sections were rewritten to improve precision, reduce ambiguity, and avoid overly broad clinical claims. The revised manuscript reflects these language and content improvements throughout.

**Reviewers' comments:

Reviewer's Responses to Questions**

**Comments to the Author**

1. If the authors have adequately addressed your comments raised in a previous round of review and you feel that this manuscript is now acceptable for publication, you may indicate that here to bypass the “Comments to the Author” section, enter your conflict of interest statement in the “Confidential to Editor” section, and submit your "Accept" recommendation.

Reviewer #3: All comments have been addressed

**Response:**

We sincerely thank Reviewer #3 for the positive evaluation of our revised manuscript. We are pleased that all previously raised comments have been adequately addressed and that the manuscript is now considered technically sound and suitable for publication. We appreciate the reviewer’s constructive feedback throughout the review process, which has significantly improved the quality and clarity of this work.

2. Is the manuscript technically sound, and do the data support the conclusions?

The manuscript must describe a technically sound piece of scientific research with data that supports the conclusions. Experiments must have been conducted rigorously, with appropriate controls, replication, and sample sizes. The conclusions must be drawn appropriately based on the data presented.

Reviewer #3: Yes

**Response:**

We thank Reviewer #3 for confirming that the manuscript is technically sound and that the data support the conclusions. We have ensured that the methodology, data analysis, and interpretation are clearly described and aligned with the presented results in the revised manuscript.

3. Has the statistical analysis been performed appropriately and rigorously?

Reviewer #3: I Don't Know

**Response:**

We thank Reviewer #3 for this important observation. We would like to clarify that this study does not rely on inferential statistical analysis, as the primary objective is to develop and evaluate an algorithm-assisted signal-processing framework for interpreting voltammetric data.

The analysis is based on deterministic electrochemical signal-processing methods, including baseline correction and peak detection, which are standard approaches in voltammetric analysis. The extracted parameter—peak-to-baseline current response—is a direct analytical measure of electrochemical activity and does not require statistical inference for interpretation.

To improve clarity, we have revised the manuscript to explicitly state the analytical approach and its rationale in the Materials and Methods and Results and Discussion sections. These revisions clarify that the study focuses on reproducible signal extraction and comparative evaluation with reference software (PSTrace), rather than statistical hypothesis testing.

4. Have the authors made all data underlying the findings in their manuscript fully available?

The [PLOS Data policy](http://www.plosone.org/static/policies.action#sharing) requires authors to make all data underlying the findings described in their manuscript fully available without restriction, with rare exception (please refer to the Data Availability Statement in the manuscript PDF file). The data should be provided as part of the manuscript or its supporting information, or deposited to a public repository. For example, in addition to summary statistics, the data points behind means, medians and variance measures should be available. If there are restrictions on publicly sharing data—e.g. participant privacy or use of data from a third party—those must be specified.

Reviewer #3: Yes

**Response:**

We sincerely thank Reviewer #3 for confirming that the data underlying the findings are fully available and comply with the journal’s data-sharing requirements.

In the revised manuscript, we have ensured that all relevant data used in this study—including voltammetry measurement datasets and processed analytical outputs—are accessible in accordance with the PLOS Data Availability policy. The Data Availability Statement has been clearly specified to indicate how the data can be accessed without restriction.

We believe that this transparency supports reproducibility and enables further validation and reuse of the proposed algorithm-assisted framework.

5. Is the manuscript presented in an intelligible fashion and written in standard English?

PLOS ONE does not copyedit accepted manuscripts, so the language in submitted articles must be clear, correct, and unambiguous. Any typographical or grammatical errors should be corrected at revision, so please note any specific errors here.

Reviewer #3: Yes

**Response:**

We thank Reviewer #3 for confirming the clarity of the manuscript. The language has been further refined to ensure consistency, readability, and compliance with standard scientific English.

6. Review Comments to the Author

Please use the space provided to explain your answers to the questions above. You may also include additional comments for the author, including concerns about dual publication, research ethics, or publication ethics. (Please upload your review as an attachment if it exceeds 20,000 characters)

Reviewer #3: (No Response)

**Response:**

We thank Reviewer #3 for the evaluation of our manuscript. As no additional comments were provided, no further response is required.

7. PLOS authors have the option to publish the peer review history of their article ([what does this mean?](https://journals.plos.org/plosone/s/editorial-and-peer-review-process#loc-peer-review-history)). If published, this will include your full peer review and any attached files.


If you choose “no”, your identity will remain anonymous but your review may still be made public.


**Do you want your identity to be public for this peer review?** For information about this choice, including consent withdrawal, please see our [Privacy Policy](https://www.plos.org/privacy-policy).

Reviewer #3: No

**Response:**

We respect the reviewer’s decision to remain anonymous and appreciate their time and effort in evaluating our manuscript.
